# Putting a new spin on insect jumping performance using 3D modeling and computer simulations of spotted lanternfly nymphs

**DOI:** 10.1242/jeb.246340

**Published:** 2023-10-06

**Authors:** Chengpei Li, Aaron J. Xu, Eric Beery, S. Tonia Hsieh, Suzanne Amador Kane

**Affiliations:** ^1^ Physics and Astronomy Department, Haverford College, Haverford, PA 19041, USA; ^2^ Department of Biology, Temple University, Philadelphia, PA 19122, USA

**Keywords:** Locomotion, Biomechanics, Tumbling, Maneuverability, Invertebrate, Antipredator behavior

## Abstract

How animals jump and land on diverse surfaces is ecologically important and relevant to bioinspired robotics. Here, we describe the jumping biomechanics of the planthopper *Lycorma delicatula* (spotted lanternfly), an invasive insect in the USA that jumps frequently for dispersal, locomotion and predator evasion. High-speed video was used to analyze jumping by spotted lanternfly nymphs from take-off to impact on compliant surfaces. These insects used rapid hindleg extensions to achieve high take-off speeds (2.7–3.4 m s^−1^) and accelerations (800–1000 m s^−2^), with mid-air trajectories consistent with ballistic motion without drag forces or steering. Despite rotating rapidly (5–45 Hz) about time-varying axes of rotation, they landed successfully in 58.9% of trials. They also attained the most successful impact orientation significantly more often than predicted by chance, consistent with their using attitude control. Notably, these insects were able to land successfully when impacting surfaces at all angles, pointing to the importance of collisional recovery behaviors. To further understand their rotational dynamics, we created realistic 3D rendered models of spotted lanternflies and used them to compute their mechanical properties during jumping. Computer simulations based on these models and drag torques estimated from fits to tracked data successfully predicted several features of the measured rotational kinematics. This analysis showed that the rotational inertia of spotted lanternfly nymphs is predominantly due to their legs, enabling them to use posture changes as well as drag torque to control their angular velocity, and hence their orientation, thereby facilitating predominately successful landings when jumping.

## Teaser

**Summary:** High-speed video shows that juvenile spotted lanternflies are adept at landing after tumbling rapidly mid-air during jumping; computer simulations and realistic 3D models are presented to help explain these abilities.

## INTRODUCTION

Jumping is an energetically costly mode of locomotion, requiring large force generation and challenging levels of sensorimotor control (Biewener and Patek, 2018). Animals jump to escape predators, capture prey and gain access to resources, traverse complex environments and navigate obstacles. Because jumping can be critical for survival, it is employed among a wide diversity of animal taxa across a broad range of sizes (Hawkes et al., 2022; Mo et al., 2020b). Biomechanical studies that determine how animals accomplish such demanding feats are therefore both ecologically relevant and a significant source of inspiration for the design of jumping robots (Mo et al., 2020b; Ribak, 2020). Motions during jumping can be broken into three phases: take-off, mid-air, and impact and landing. Most prior studies have focused on the initial take-off period most critical for achieving jumps with optimal values of height and horizontal jumping distance (e.g. Burrows et al., 2021; Mo et al., 2020b; and references therein). However, jumping also presents the organism with challenges in controlling both the direction of its jump and its body orientation throughout the trajectory. Therefore, some recent studies have considered how the mid-air phase is not simply a passive period of parabolic flight, but rather can include active and passive stabilization measures, as well as directed navigation critical to a controlled landing (Burrows et al., 2015; Ortega-Jimenez et al., 2022; Zong et al., 2022).

Among insects, planthoppers (infraorder Fulgoromorpha) in particular have exceptional jumping abilities that have been studied extensively in the preparatory and take-off phases (Burrows, 2009, 2014a; Burrows et al., 2019). Although these insects make extensive use of jumping, it is less well documented how they manage the mid-air and landing phases. The spotted lanternfly (*Lycorma delicatula*) is a species of planthopper that has emerged recently as a highly invasive pest in the USA and South Korea, posing a significant agricultural threat. Spotted lanternflies jump as a dominant form of locomotion to disperse, find food and habitat, and evade predators (Nixon et al., 2021). Like many other insects, they also need to be able to land on complex landscapes formed by densely clustered leaves oriented at random, unpredictable angles (Graham and Socha, 2020; Kane et al., 2021; Zeng et al., 2020). In this study, we present the first exploration of spotted lanternfly jumping biomechanics. Our interdisciplinary approach utilized empirical data on jumping kinematics using high-speed 3D video, detailed 3D rendered models, and computer simulations of the resulting trajectories and rotational dynamics. In addition to characterizing their jumping performance at take-off, we also considered how these insects negotiate the post-take-off mid-air and landing phases. We hypothesized that spotted lanternflies would extend their legs during the mid-air phase of a jump to decrease the body's angular speed and increase the probability of a successful landing.

In particular, we studied whether spotted lanternfly nymphs utilize several mid-air behaviors observed for other insects. First, we considered directed aerial descent, in which jumping and falling insects and spiders change their trajectory direction in mid-air (Yanoviak et al., 2010, 2011, 2015; Zeng et al., 2015) to enhance their ability to land on an intended target (Socha et al., 2015). To test the hypothesis that spotted lanternfly nymphs might use directed aerial descent, we measured whether their tracks while in the air deviated from a planar ballistic trajectory, and whether the timing of changes in their posture correlated with changes in body orientation, heading, speed and angular velocity (Yanoviak et al., 2010, 2015).

Second, we studied whether nymphs can reorient their bodies while in the air (attitude control), to facilitate landing. Such aerial reorientation can be a passive consequence of aerodynamic drag and body posture, as found during falling for dragonflies (Fabian et al., 2021), pea aphids (Ribak et al., 2013), spiders (Yanoviak et al., 2015) and spotted lanternfly nymphs (Kane et al., 2021), and during jumping for springtails (Ortega-Jimenez et al., 2022), or can involve active body motions [e.g. stick insect nymphs (Zeng et al., 2017), mantises (Burrows et al., 2015), lizards (Higham et al., 2017; Jusufi et al., 2008; Siddall et al., 2021), frogs (Wang et al., 2022) and squirrels (Fukushima et al., 2021)]. We therefore studied whether and how spotted lanternfly nymphs attain different body postures at different phases during jumping, particularly before landing attempts.

Third, we investigated whether spotted lanternfly nymphs rotate rapidly after take-off, as has been observed for several other insect species (Brackenbury, 1996; Burrows, 2012; Burrows et al., 2007, 2015; Goode and Sutton, 2023; Zong et al., 2022). Such behaviors are ecologically salient because mid-air rotational motion can complicate self-righting, targeting and landing, as well as potentially generate lift and thereby extend the aerial trajectory via the Magnus effect (Wauthy et al., 1998), and enhance predator evasion (Burrows and Dorosenko, 2014). Several recent studies have explored how spinning insects can reduce their angular velocity and reorient in mid-air to facilitate landing upright by bending their flexible bodies (Ortega-Jimenez et al., 2022) or deploying their wings (Zong et al., 2022). To determine whether spotted lanternfly nymphs indeed rotate during jumping, and whether they use changes in body posture or drag torques to control their angular velocity and orientation, we used video tracking data to measure their rotational motion during jumping, and to test for correlations between angular velocity and changes in body posture. To interpret the video data, we expanded on earlier studies that modeled elongated insects as assemblies of jointed rods (Burrows et al., 2015; Zeng et al., 2017) by creating detailed 3D rendered models of the insect's body in postures observed during various jumping phases, and used these to parameterize computer simulations of their rotational dynamics.

**List of symbols and abbreviations**

*a*_TO_: acceleration during take-off
COM: center of mass
*e*_ballistic_: ballistic fit residuals
*e*_planar_: planar fit residuals
*F*: force
*I*_rot_: moment of inertia
*I_i_*: moment of inertia along the *i*th principal axis
*L*: angular momentum
*L*_b_: body length
*r*: moment arm magnitude
*R*: maximum jumping distance
*Re*: Reynolds number
*S*_o_: body-fixed frame of reference with origin at the object's COM
*S*_s_: spatial (inertial) frame of reference
*t*_accel_: acceleration time for take-off
*t*_char_: characteristic rotational drag time scale
*t*_ext_: time from take-off until the spotted lanternfly extends its legs fully
*T*_osc_: oscillation period
*t*_TOF_: time of flight
û_i_: unit vector lying along the *i*th principal axis
*v*_TO_: take-off speed
θ: take-off angle relative to horizontal
μ: dynamic viscosity of air
ρ: air density
τ: torque (moment of force)
ω: angular velocity
ω_cc_: angular speed of the cranial–caudal axis
ω_ext_: ω_cc_ when legs are first fully extended
ω_TO_: ω_cc_ immediately after take-off

## MATERIALS AND METHODS

### Insect methods

**Fig. 1.**
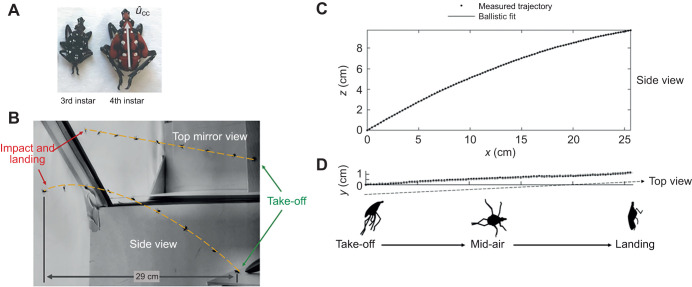
**Laboratory jumping experiments.** (A) Spotted lanternfly nymph third and fourth instars. Gray arrow shows the cranial–caudal axis, 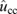, which was tracked to measure body orientation versus time. (Adapted from Bien et al., 2023.) (B) Jumping experiment arena photograph showing the launch pad (bottom right), superimposed images of a spotted lanternfly nymph at different times and the jumping trajectories (dashed yellow line), and the white fabric target (far left) on which they impacted and landed, as seen in the main camera side view and orthogonal top mirror view. (C,D) Spotted lanternfly nymph jumping raw trajectory coordinate data (circles) and 3D ballistic fits (lines) as seen from the (C) side and (D) top views. Here, *z* is the vertical, *x* and *y* are in the horizontal plane.

Third and fourth instar spotted lanternfly nymphs, *Lycorma delicatula* (White 1845) (Fig. 1A) were collected between mid-morning and late afternoon (Kim et al., 2011) from *Ailanthus altissima* (tree-of-heaven) and *Vitis vinifera* (wild grape vines) in southeastern Pennsylvania (40°00′30.2″N, 75°18′22.0″W) in late June to early July 2021. Specimens were maintained on the leaves of potted ≥1 m tall *A. altissima*, their preferred native host plant, as recommended for raising spotted lanternflies in the laboratory (Nixon et al., 2022), and used for experiments within 2 h of collection. For this purpose, *A. altissima* trees were collected from the field with intact root systems, maintained in mesh cages under full-spectrum grow lights, and replaced before they began to show stress from spotted lanternfly feeding. After experiments were completed, the body mass of each specimen was measured and used to determine life stage for the third instars (Bien et al., 2023). Laboratory environmental conditions were maintained at 23°C (22–24°C) and 64% (44–73%) relative humidity (mean, full range).

### Data analysis and statistics

All fitting and statistical analyses were performed in MATLAB vR2022a (MathWorks, Natick, MA, USA) and R (http://www.R-project.org/); all mentions of MATLAB function calls are italicized. Hypothesis testing was performed using a significance level of 0.05. Data are reported as grand means [95% confidence interval, CI] computed from means of individual specimen means, unless stated otherwise. We use *N* to denote the number of individual specimens and *n* for number of trials.

### Jumping experiments

Two preliminary field studies were performed to aid in the design of laboratory experiments. To determine whether jumping behaviors in the laboratory resembled those in the field, spotted lanternfly fourth instar nymphs were filmed jumping and landing on leaves, either spontaneously or in response to being prodded by a finger, using a GoPro Hero 9 Black Edition (1920×1080 pixels, 240 frames s^−1^); only six videos of six different individuals were obtained because of the challenges inherent in filming small insects in dense foliage. In addition, to check for fatigue due to repeated jumping, we observed fourth instar spotted lanternfly nymphs (*n*=6 trials, 1 trial per specimen) that were placed on a stone walkway in the shade and then stimulated to jump toward the sunlit bed of foliage upon which they were collected. The starting and end position of each jump was marked and their distance measured to determine jumping distance. Linear regression performed on the data found weak correlation between jumping distance (85±16 cm, mean±s.e.m.) and jump number for all specimens (*R*^2^≤0.22), with only one of the six specimens having a significant dependence between jumping distance and jump number (*P*=0.042). (Fig. S1, Table S1). This supported studying multiple jumping trials by individual specimens in the laboratory experiments.

In the laboratory, we recorded high-speed 3D video for spotted lanternfly nymphs jumping from a raised platform to a compliant vertical target, a geometry chosen to resemble jumping between different locations in plants while providing an unimpeded view of the insect. The horizontal 16.5 cm deep×20.5 cm wide×23 cm high take-off platform was made of 80-grit sandpaper spray-painted white and mounted on hardboard; this rough substrate was used because the specimens avoided jumping from smoother surfaces (e.g. posterboard or glass), instead electing to walk away or climb to the underside of the platform. The jumping experiment arena (Fig. 1B) had white acrylic and posterboard walls on two sides, a clear Plexiglas wall for observing and positioning specimens, and white insect netting to reduce air currents and prevent escapes. Previous research indicated this species exhibits positive phototaxis (Domingue and Baker, 2019; Jang et al., 2013), so specimens were released in front of a brightly backlit white mesh insect netting placed over the white fabric cover of the LED light source used to illuminate the scene for videography; the netting also served as a compliant surface that the nymphs were able to grasp and cling to. The horizontal distance from the take-off platform to the target was approximately 29 cm, a value chosen as approximately half the mean horizontal jumping distance found for third and fourth instar spotted lanternfly nymphs (Nixon et al., 2021).

Videos were filmed using a single Chronos 1.4 video camera (1280×1024 pixels; 1000 frames s^−1^, 200 μs exposure time) that captured the trajectory in two orthogonal views: a side view in the approximate plane of the trajectory, and an overhead view reflected in a mirror oriented at 45 deg to the horizontal (image resolution: 3.6 and 3.0 pixels mm^−1^, respectively). The arena was illuminated by a Godox white LED lamp at intensities (22,100±100 lx) similar to values measured in the open areas surrounding trees where spotted lanternflies were collected.

To induce spotted lanternfly nymphs to jump, specimens were placed gently onto the take-off platform using scoop-shaped forceps and allowed to move freely. If a nymph oriented toward the target, it was encouraged to jump by approaching it rapidly with a finger or pen to simulate predator attack. The jumping strategies explored here therefore likely reflect escape behaviors. When possible, specimens were recaptured and induced to jump for repeated trials in the same session.

Experiments were performed with the goal of obtaining at least 15 videos for the two nymphal life stages studied that showed the entire jump trajectory in both camera views from take-off to either successful or attempted and failed landing (‘analyzable’ videos). All videos recorded were viewed repeatedly to find those that matched these criteria and then analyzed for jumping performance. Videos that showed at least the body orientation at impact and the outcome (successful or failed landing attempts) were separately scored to study behavior during attempted landing.

### Video analysis and trajectory fitting

All analyzable videos were scored manually to determine the postures assumed during jumping, the time required to assume each posture, the time of first impact, landing orientation and strategy, and whether the specimen landed successfully. The phases during jumping were defined as take-off (when leg motions were first evident to when the feet first lost contact with the surface), mid-air (take-off to first impact), impact (when the body first contacted a surface) and landing. A successful landing was defined as an impact in which the spotted lanternfly nymph was able to cling to the surface, and a failed landing as one in which the spotted lanternfly nymph either bounced off or fell off the target surface. The orientation at impact was estimated from the closest match between the landing surface and the body ventral, dorsal, right, left, cranial or caudal surfaces. To test for dependence of landing success on orientation at impact, we compared our measured proportions with a simple model of equal probabilities (1/6) of landing in each of the six orientations using MultinomCI (http://www.R-project.org/) to compute the 95% CI for the measured proportions for each outcome.

The spotted lanternfly nymph body center of mass (COM) was tracked throughout each trial on both camera views using a combination of custom MATLAB code and semi-automatic tracking using the program Direct Linear Transformation data viewer (DLTdv) (Hedrick, 2008). A combination of DLTdv and 3D reconstruction parameters determined by wand calibration (Theriault et al., 2014) [wand score of 0.6, wand end point s.d.=0.4 mm (0.4% span), and pixel projection errors 0.11–0.15 pixels ≤0.54 mm] was used to convert the 2D tracked coordinates on the two camera views into 3D coordinates with the origin at the point of take-off, *z* oriented vertically upward (as determined by a freely falling ball), and *x* and *y* in the horizontal plane (Fig. 1C). The tracked coordinates versus time were used to compute the velocity direction (heading) using quadratic polynomial regression over 25 ms time windows; this method resulted in a root-mean-square (rms) error between the fit and measured track data equal to the 3D reconstruction uncertainty.

The raw 3D trajectories from take-off to impact were fitted to a zero-drag ballistic model using non-linear least squares regression, an approach used to characterize the kinematics of jumping performance of spiders and crickets (Nabawy et al., 2018; Palmer et al., 2018), using the equation for a parabola:
(1)

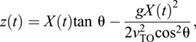
where *X*(*t*) is distance from the position at take-off projected onto the horizontal *x–y* plane (Fig. 1C), *v*_TO_ is take-off speed, *g*=9.81 m s^−2^ is the acceleration of gravity, and θ is take-off angle with respect to the horizontal (Taylor, 2005). All fits to Eqn 1 were performed allowing all parameters except *g* to vary freely.

We used the results of fitting to look for three effects that can cause the measured trajectories to deviate from the simple zero-drag ballistic model. First, drag forces can cause the trajectory to deviate systematically from a parabola at longer times. Second, body rotations and changes in posture can cause the trajectory of the whole insect COM to deviate from that for the body-only COM, which was tracked on video; this can result in non-random variations in the fit residuals and heading angles. Third, while a zero-drag ballistic trajectory lies in a vertical plane, steering can cause variations in the azimuthal heading angle (direction of the velocity's horizontal component), and hence a non-planar trajectory. To test for steering, the 3D coordinates of each trajectory were also fitted to a plane using *pcfitplane*. To test for these three effects, we assessed the goodness-of-fit of the zero drag force, no-steering model by comparing the residuals for the ballistic trajectory fit, *e*_ballistic_ (Eqn 1) and the planar fit, *e*_planar_ to the 3D reconstruction uncertainty. Plots of these fit residuals versus time also were examined for non-random variation by comparing the rms values of the residuals (RMSE) for the ballistic and planar fits between three time intervals: (1) within 25 ms of take-off (*e*_take-off_); (2) within 25 ms of mid-air posture changes (*e*_mid-air_) and (3) within 5 ms of impact (*e*_impact_).

Jumping performance was characterized by computing the acceleration during take-off using *a*_TO_=*v*_TO_/*t*_accel_ (where *t*_accel_ is acceleration time). The maximum jumping distance, *R*, and time-of-flight, *t*_TOF_, corresponding to fitted values of *v*_TO_ and the optimal take-off angle θ=45 deg, were estimated using *R*=*v*_TO_^2^sin^2^θ/*g* and *t*_TOF_=2*v*_TO_sinθ/***g*** (Taylor, 2005). We also characterized the aerodynamic regime during jumping by computing the Reynolds number, *Re*=ρ*v*_TO_*L*/μ, with air density ρ=1.2 kg m^−3^, body length *L*_b_=9.3 and 11.7 mm for third and fourth instar spotted lanternfly nymphs, respectively, and the dynamic viscosity of air μ=18.3×10^−6^ Pa s (Alexander, 2016).

### Measuring and simulating rotational kinematics and dynamics

Spotted lanternfly nymph body orientation and rotational kinematics during jumping were tracked manually on video in 3D using DLTdv; results are reported relative to a coordinate frame, *S*_s_ (the ‘spatial frame’) with its axis directions fixed relative to the laboratory and its origin at the nymph's COM. Spotted lanternfly nymph angular orientation and velocity were described using the Tait–Bryan (roll–pitch–yaw) angle convention in *ZYX* order, using the orientation of the body principal axes of rotation defined below relative to the *xyz* axes in the laboratory defined above. The initial 3D angular velocity vector, **ω**(0), was computed using the MATLAB function *angvel* with the 3D orientation of the nymph immediately after take-off and 5 ms later. Nymphs were not resolved well enough on video for the entire jumping trajectory to allow tracking of their full 3D orientation on every frame and thus to enable the calculation of the full angular velocity vector. Instead, we found the orientation of their cranial–caudal axis, **u**_cc_, for the entire trajectory by tracking one point on the head and one on the caudal end on each frame from initial take-off until impact on the landing target, and then using custom MATLAB code to compute the angular speed for rotations of the cranial–caudal axis using: ω_cc_(*t*)=|**u**_cc­_/Δ*t*| (Fig. 1A). The values of ω_cc_ versus time were used to compute several measures of rotational kinematics, including angular speed at take-off, ω_TO_, and the ratio of angular speed at leg extension to that at take-off, ω_ext_/ω_TO*,*_. Because the tracked data for some trials exhibited oscillations in ω_cc_(*t*) versus time, the oscillation period, *T*_osc_, was measured manually from the time intervals between successive peaks. For consistency with earlier studies, we report ω in units of Hz, where 1 Hz=2πrad s^−1^.

**Fig. 2.**
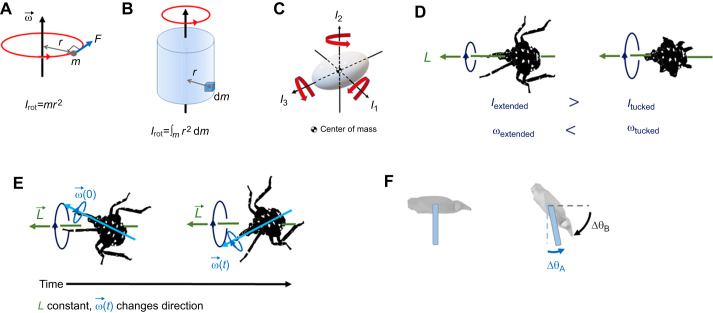
**Measuring and simulating rotational kinematics and dynamics.** (A) Illustration of the geometry used in defining the quantities used to describe rotational dynamics about a single axis for a point mass: ω, angular velocity; *r*, moment arm; *F*, force; *m*, mass; *I*_rot_, moment of inertia. (B) Geometry for computing moment of inertia, *I*_rot_, for an extended rigid body. (C) Schematic illustration of the principal axes of rotation for an extended body, labeled by their corresponding values of moment of inertia. (D) For zero external torque, angular momentum, *L*, is constant, so changes in body posture (e.g. from legs extended to legs tucked) that alter moment of inertia, *I*_rot_, also change angular velocity, ω. (E) Illustration of the geometry for torque-free precession, in which the rotational velocity vector, 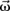, and its associated axis of rotation (blue arrows) describe a cone about the constant angular momentum vector, 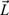 (green arrow), even if the external torque is zero. (F) Illustration of the geometry for Eqn 7 and surrounding discussion. The spotted lanternfly is modeled as a counter-rotating body (gray) and rod-like leg (blue box) with a hinge joint shown before rotation (left) and after rotation (right).

Computer simulations were used to reconstruct full 3D rotations and interpret how the mid-air rotational motions during jumping related to the observed spotted lanternfly nymph body postures. This allowed us to test hypotheses about whether these insects use attitude control. The 3D rotational dynamics during jumping was modeled using the following results from advanced classical mechanics; see Goldstein (1980) for an in-depth treatment. The equation of motion for the simplest case of a point mass, *m*, rotating about a fixed axis is:
(2)

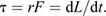
Here, τ is the net external torque (moment of force), *r* is the magnitude of the moment arm (the vector from the point mass to the axis of rotation), *F* is the component of force perpendicular to the moment arm, *t* is time, and *L* is angular momentum, *L*=*I*_rot_ω, where *I*_rot_=*mr*^2^ is the moment of inertia, a measure of resistance to changes in angular velocity, and ω is angular velocity in rad s^−1^ (Taylor, 2005) (Fig. 2A). For an extended rigid object rotating about a given axis, *I*_rot_ depends on the object's mass distribution relative to the axis of rotation (Fig. 2B) as:
(3)

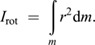


In 3D, torque and angular velocity are both vectors and the moment of inertia is a 3×3 matrix, the inertia tensor, **I**_rot_ (Goldstein, 1980). There exists a coordinate frame, *S*_o_ (the ‘object frame’) with its origin at the COM in which the object's orientation is constant and the inertia tensor is a diagonal matrix with elements *I*_1_, *I*_2_ and *I*_3_. In this frame, the coordinate axes, 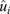, are called the object's principal axes, and *I_i_* is the moment of inertia for rotations about the *i*th principal axis (Fig. 2C).

The full 3D rotational dynamics of a rigid body in frame *S*_o_ with its origin fixed at the object's COM are given by the 3D analogy to Eqn 2, the Euler equations:
(4a)

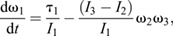

(4b)

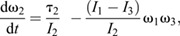

(4c)

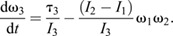


These equations were integrated to solve for the object's angular velocity vector, **ω**(*t*)=(ω_1_, ω_2_, ω_3_), using MATLAB's ordinary differential equation solver, *ode45* with a simulation timestep 2.6×10^−6^ s (≤0.04 deg per time step). The angular velocity for each time step then was converted into a sequential series of 3D rotations to give the object's rotational motion in the spatial frame, *S*_s_, using custom MATLAB code. The parameters required for solving Eqn 4 were obtained as follows (Marghitu and Dupac, 2012). As described above, the initial angular velocity, **ω**(0) was measured directly from video. The moments of inertia (*I*_1_, *I*_2_ and *I*_3_) were computed using 3D models of the spotted lanternflies, as explained in the next section. Because gravity acts on the COM and hence does not exert torque, the only external torque acting once the insect was airborne was due to aerodynamic drag, τ_drag_. We modeled the drag torque associated with rotations about the *i*th axis of rotation using:
(5a)

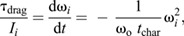

(5b)

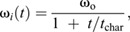


where *t*_char_ is a characteristic time for the angular velocity to decrease to half its initial value, and ω_o_=ω*_i_*(0) (Taylor, 2005). Measured values of ω_cc_(*t*) from tracking were fitted to Eqn 5b using non-linear least squares fits in MATLAB to find *t*_char_ for the legs-tucked and legs-extended poses; this was then used with Eqn 5a and ω_o_ to estimate the values of τ_drag_/*I_i_* used in solving Eqn 4.

Video analysis showed that spotted lanternfly nymphs assume characteristic body postures during jumping, and that the axis of rotation often changed during the mid-air phase even for a fixed posture. To explain this phenomenon, we note that the Euler equations allow the direction of the angular velocity to change with time even if net external torque is zero and angular momentum is constant because in 3D, angular momentum is given by **L**=**I**_rot_ · **ω**. If the insect's body has two or more different values of *I_i_*, this means that **L** and **ω** only have the same direction for rotations purely about one principal axis. If an insect with constant posture does not rotate about a principal axis, it will undergo a form of rotational motion called torque-free precession in which its angular velocity describes a cone about the constant angular momentum vector (Butikov, 2006) (Fig. 2E). We therefore computed the values of *I_i_* for the observed nymph postures to test whether this argument (i.e. unequal values of *I_i_*) could account for their observed rotations.

We also modeled how the observed changes in posture by spotted lanternfly nymphs affect their moments of inertia, and hence body orientation and angular velocity as a function of time. One possibility is that they might extend or tuck their legs, similar to when a human tucks or extends their arms and legs during a dive, so as to keep the principal axes constant but change one or more values of *I_i_* (Fig. 2D). If drag torque is negligible, then in this scenario Eqn 4 reduces to *L_i_*=*I_i_*ω*_i_*=constant (angular momentum conservation) along each principal axis, and the moments of inertia and angular velocity for the two postures then are related as:
(6)

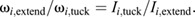
Eqn 6 explains how divers can spin faster by tucking their arms and legs (increasing ω*_i_* by decreasing *I_i_*) or spin more slowly by extending them (decreasing ω*_i_* by increasing *I_i_*). More generally, changes in body posture can alter the body's orientation and rotational axis, not only its angular speed. For example, this occurs when a diver ‘throws’ one or more appendages (i.e. rapidly rotates arms or legs across the body) during a dive. If this change in posture also reorients the principal axes, it will change the direction of **ω** even though angular momentum is constant. This can be understood using an articulated model in which the body (B) and appendages (A) can rotate relative to each other, such that total angular momentum, **L**=**L**_B_+**L**_A_. The throw can be interpreted as exchanging angular momentum between the body and appendages (A) such that the change in **L**, Δ**L** is zero and hence Δ**L**_B_=−Δ**L**_A_. For this reason, the resulting motion is called an angular momentum twist. Competition divers use this effect to execute twisting dives in which, for example, they initially rotate by pitching, perform a throw to execute a complete roll, then execute a second throw so as to resume pitching (Frohlich, 1979). Sequential application of this mechanism also allows animals to achieve a preferred orientation mid-air, e.g. falling cats can use it to self-right aerially (see Frohlich, 1980, for a non-mathematical discussion). A simple model of this can be constructed by considering that the object consists of two jointed rigid segments with moment of inertia *I*_A_ and *I*_B_ oriented by a relative angle θ along a principal axis. If segment A rotates by Δθ_A_ over time d*t*, then B must counter-rotate to conserve angular momentum, resulting in a body rotation of Δθ_B_ (Fig. 2F):
(7a)

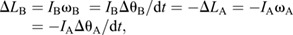

(7b)

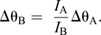
After this maneuver, the insect can maintain this body rotation, either by keeping the legs fixed or by tucking them close to the body without changing angular momentum. Previous studies have combined video tracking and modeling to show how angular momentum exchange influences aerial reorientation by insects with rod-like bodies and legs, e.g. jumping mantises and falling stick insect instars (Burrows et al., 2015; Zeng et al., 2017). Here, we modeled how the observed changes in spotted lanternfly nymph posture during jumping affect both *I_i_* and the principal axis direction, and therefore the rotational motions predicted by Eqn 4.

### 3D modeling and calculation of mechanical properties

**Fig. 3.**
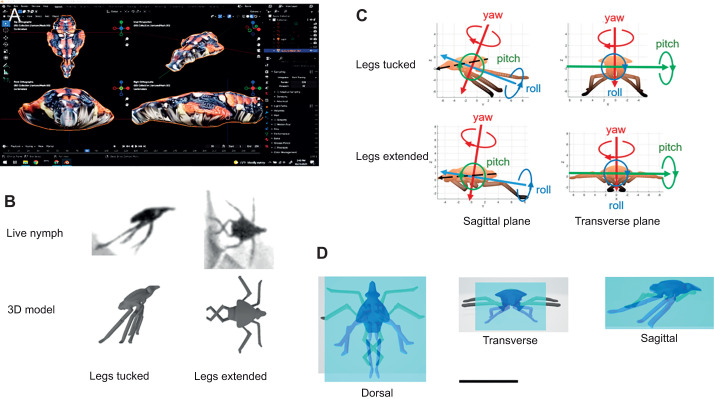
**3D rendered models of spotted lanternfly nymphs.** (A) Photographs of specimens taken from multiple views were used with photogrammetry software to create detailed 3D renderings of spotted lanternfly nymph bodies and legs. (B) Video images of a live spotted lanternfly nymph and the 3D models for two stereotyped mid-air postures observed during jumping. (C) Orientation of the principal axes for the two 3D models, showing the definition of the roll, pitch and yaw axes used in this study. (D) Anatomical plane views used for estimating 3D model cross-sectional areas in Table 3. The images of the legs-tucked (blue) and legs-extended (gray) 3D models in each body plane are overlaid to allow a visual comparison of their relative cross-sectional areas. Scale bar: 10 mm.

Realistic 3D models of spotted lanternfly nymphs were created for calculating their mechanical properties for use in computer simulations. First, reference images were recorded using freshly euthanized spotted lanternfly nymphs mounted on a size 00 insect pin and photographed from multiple angles using a Sony DSC-RX100 camera in macro mode under diffuse LED lighting. Photographs were obtained at five different camera elevation angles for every 10 deg of horizontal rotation using a turntable to rotate the specimen. The resulting photos were then analyzed by photogrammetry using Meshroom, an open-source 3D reconstruction program (https://alicevision.org/#meshroom, accessed 16 January 2023) to create a 3D mesh of the photographed surface (Fig. 3A). Following methods outlined in Semple et al. (2019), the 3D mesh and the measured body and leg dimensions were used with the open-source 3D graphics package Blender (www.blender.org, accessed 16 January 2023) as references for creating a detailed, fully rigged (i.e. articulated) 3D model (Fig. 3B). The Blender model's accuracy was checked by using it to create and measure the leg and body dimensions of a 3D printed physical model to confirm that they agreed with those measured for actual spotted lanternfly nymphs. Next, the 3D model for each specific spotted lanternfly nymph posture was created by treating the body as a rigid object while configuring the legs to agree with the joint angles observed for the live insect on video. Each 3D model was used to create a .stl file that contains the triangular mesh describing the surface geometry of the model corresponding to each posture.

The MATLAB package RigidBodyParams (https://github.com/AntonSemechko/Rigid-Body-Parameters) was used to compute the volume and COM coordinates for each 3D model, assuming constant density and a body length, *L*_b_=8.9 mm and mass 28.4 mg typical of third instar spotted lanternfly specimens. As a check on the accuracy of the 3D model in describing the insect's body morphology, we used the model's volume to compute an effective body density of 1.0 g cm^−3^, which agrees with published values for insects (Kühsel et al., 2017). The values of **I**_rot_=(*I*_1_, *I*_2_, *I*_3_) and the principal axes used for solving Eqn 4 also were computed using RigidBodyParams and 3D models of spotted lanternfly nymph postures observed on video during different phases of jumping. (Fig. 3C). To interpret how drag forces might vary with posture and orientation relative to airflow, we used ImageJ (Schneider et al., 2012) to find the planar cross-sections of each model (Fig. 1D), which correspond to the drag reference areas for intermediate to high Reynolds number (Vogel, 2020).

Because this study was designed to understand the effect of the major changes in posture observed (e.g. extent and angle of leg extension), we used one 3D model for each stereotyped posture observed in our calculations and simulations. Spotted lanternfly third and fourth instar nymphs share a similar body morphology and their masses scale approximately isometrically with body length, *L*_b_ (Bien et al., 2023); these 3D models and associated mechanical properties therefore should be reasonable approximations to both life stages when scaled appropriately.

## RESULTS

### Jumping experiments

**Fig. 4.**
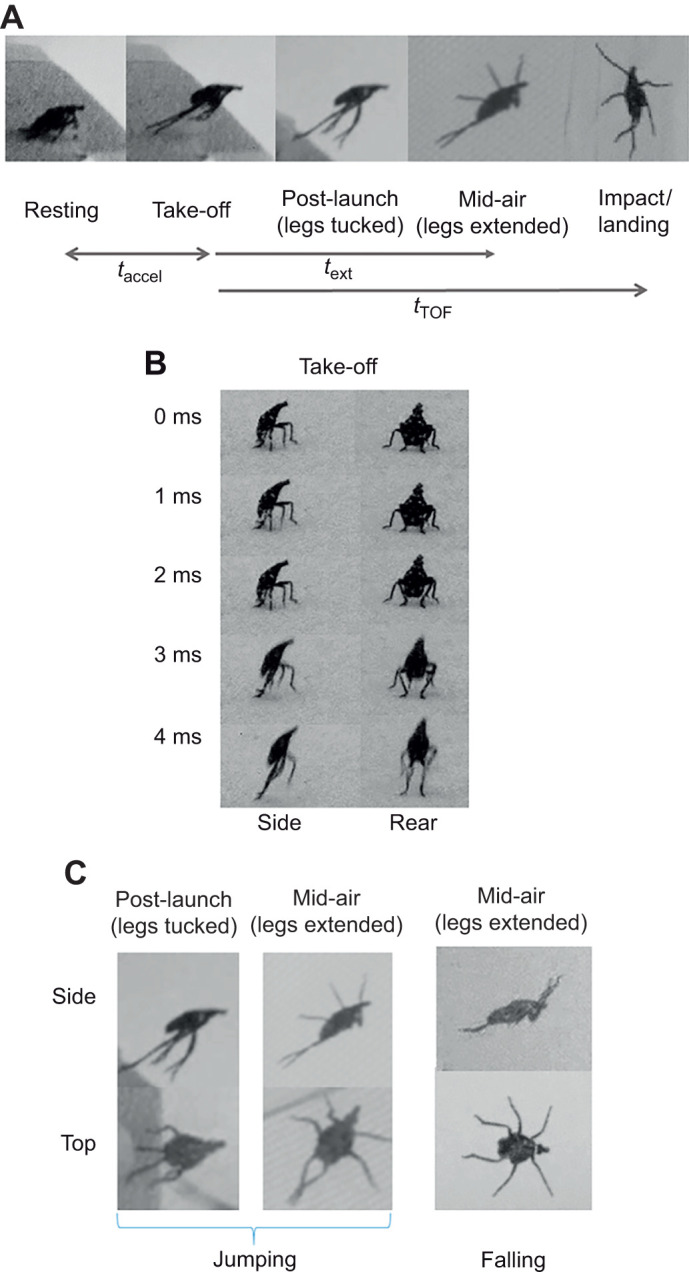
**Jumping behaviors.** (A) Ethogram of spotted lanternfly nymph jumping behavior divided into observed phases and stereotyped postures, with the time for acceleration during take-off, *t*_accel_, leg extension after take-off, *t*_ext_, and time of flight, *t*_TOF_, indicated. (B) Leg motions during take-off. (C) Stereotyped postures assumed in mid-air during jumping (this study) and falling (Kane et al., 2021).

We recorded analyzable videos of jumping for *n*=83 total trials (*N*=48 specimens; 1–5 trials per specimen) with successful landings (Table 1). In addition, we filmed 15 trials of jumping that did not show a successful landing (third instars: *N*=*n*=1; fourth instars: *N*=13, *n*=14). The ethogram in Fig. 4A summarizes the phases and associated stereotyped postures observed during jumping behaviors by spotted lanternfly nymphs: (1) take-off (Fig. 4B) – the insect first assumes a crouching posture, then rapidly extends its hindlegs to provide propulsion, as previously reported for other planthoppers (Burrows, 2009); (2) post-launch (Fig. 4C) – the insect keeps its legs tucked close to its body to varying degrees; (3) mid-air (Fig. 4C) – the forelegs and mid-legs are fully extended laterally, while the hindlegs are extended rearward and crossed; (4) impact and landing or failure to land – the insect assumes a variety of postures.

**Table 1.**
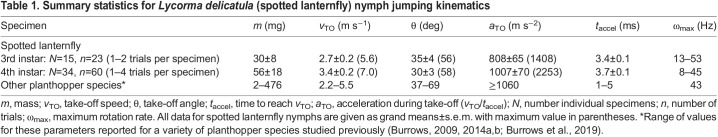
Summary statistics for *Lycorma delicatula* (spotted lanternfly) nymph jumping kinematics

### Kinematics of the jumping trajectories

Fig. 1C shows plots typical of the measured 3D jumping trajectory coordinates and the corresponding zero drag force ballistic fits for the entire trajectory to Eqn 1 for all 83 trials that showed successful landings. The complete trajectory from take-off to impact was well described by fits to this model with no contributions from drag forces or steering: (1) the values of *R*^2^ were consistently high (≥0.96); (2) the mean RMSE *e*_ballistic_ from the in-plane ballistic fit (0.39–0.43 mm) and *e*_planar_ for the best-fit plane (0.45–0.48 mm) were both close to the 3D reconstruction error (0.54 mm) (see Table S2 for full fit statistics). To characterize the kinematic parameters for jumping performance at take-off, we also performed a second set of fits to Eqn 1 for only the first 25 ms after take-off (Table 1). The fitted take-off speeds and angles were used to compute the theoretical maximum jumping distance for fourth instars of 89 cm [74, 103 cm], which agreed with measured values [86 cm [70, 102 cm] from this study and 84 cm [79, 89 cm] from Nixon et al. (2021)], while the value estimated for third instars, 66 cm [54, 77 cm], was greater than measured values (46 cm [43, 49 cm]) reported in Nixon et al. (2021). The Reynolds numbers (9.3×10^2^≤*Re*≤6.6×10^3^) computed from the take-off speeds fell within the intermediate regime (10^2^≤*Re*≤10^4^) found for other jumping and flying insects (Bennet-Clark, 1980; Ellington, 1991). Interestingly, the take-off speeds for spotted lanternfly nymphs were similar to the 2.7 m s^−1^ [2.5, 2.9 m s^−1^] terminal speed for falling fourth instars (Kane et al., 2021).

As an additional test for evidence of steering, the difference in the total fit residuals was computed before and after leg extension, and after leg extension and immediately before impact, to look for patterns that would indicate changes either in direction or in angular velocity. No significant differences were found in either case (Table S2).

Shortly after take-off, all but one of the spotted lanternfly nymphs filmed assumed a stereotypical mid-air posture (‘legs-extended’) in which the forelegs and mid-legs were extended in the transverse plane and the hindlegs crossed and extended rearward (Table 1; Movie 1). The time from take-off to full leg extension, *t*_ext_, was 40 ms [36, 44 ms] for third instar nymphs and 48 ms [45, 51 ms] for fourth instar nymphs. Specimens maintained this crossed-leg posture even after impact and landing in 12/23 trials for third and 21/61 trials for fourth instar nymphs (Movie 1). No other typical changes in posture were observed during the mid-air phase of jumping.

### Rotational kinematics

All 98 analyzable videos recorded in the laboratory and 6 field videos showed specimens rotating after take-off with a variety of initial angular velocity directions and subsequent rotational motions. The angular speed of the cranial–caudal axis, ω_cc_, versus time was tracked and analyzed for a total of 21 arbitrarily selected trials (*N*=20, 1–2 trials per specimen) (Fig. S2). After an initial analysis found no statistical differences between the rotational kinematic measures for third and fourth instars, the results were pooled across life stages (Table 2). Although the jumping trajectories were consistent with zero drag force, all plots of angular speed versus time showed a monotonic decrease consistent with aerodynamic drag torque. To provide an estimate of the rotational drag torque time scale to use in simulations, the data for ω_cc_ versus time were also fitted to Eqn 5b, giving *t*_char_=155 ms [137, 174 ms] for the legs-tucked and *t*_char_=76 ms [60, 92 ms] for the legs-extended postures (Fig. S2; Table 2). (We fitted to a single value of *t*_char_ for each posture because the orientation of the rotating nymphs constantly changed relative to the airflow direction.)

**Table 2.**
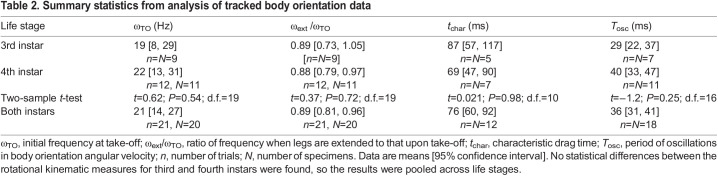
Summary statistics from analysis of tracked body orientation data

Next, the estimated moments of inertia, *I_i_*, and cross-sectional areas (Table 3) and principal axis directions (Fig. 3C) were computed for the legs-tucked and legs-extended 3D models for use in rotational dynamics simulations. While the transverse (pitch) axis agreed with the 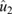
principal axis for both 3D models, the other two principal axes only approximately agreed with the body cranial–caudal and dorsal–ventral axes. Using the principal axis in closest agreement with those latter body axes, we defined roll as rotations about principal axis 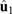 with moment of inertia *I*_1_, and yaw as rotations about 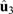
with moment of inertia *I*_3_.

**Table 3.**
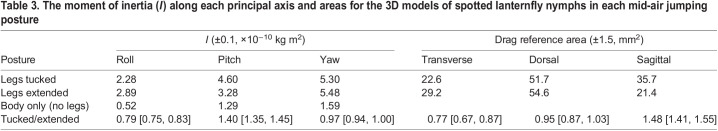
The moment of inertia (*I*) along each principal axis and areas for the 3D models of spotted lanternfly nymphs in each mid-air jumping posture

**Fig. 5.**
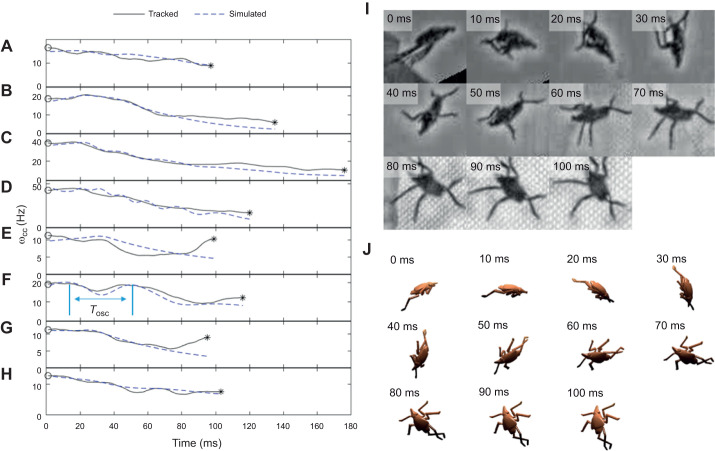
**Rotational kinematics.** (A–H) Angular velocity of body orientation (cranial–caudal axis), ω_cc_, versus time tracked on video and from simulations using parameters measured from the tracked data from take-off (circles) to impact (asterisks). Details of the simulations are described in Materials and Methods. F shows an example of how the oscillation period, *T*_osc_, was measured. (I,J) Image sequence from the video (I) and matching simulations (J) for the data in E; the spotted lanternfly model is shown in the legs-extended posture for all frames.

Given that both mid-air postures have 3 significantly different moments of inertia (Table 3), the direction of the axis of rotation is expected to vary in time unless the insect manages to take-off rotating purely around a single principal axis. Furthermore, even if the insect manages to achieve stable rotations about a principal axis (e.g. by purely pitching), the fact that the cranial–caudal axis, **u**_cc_, is not a principal axis means that its associated angular speed, ω_cc_, should vary in time due to precession. In agreement with this expectation, most plots of the measured ω_cc_ versus time displayed oscillations (Fig. 5; Fig. S2, Movie 1). The mean period for the analyzed tracks was *T*_osc_=36 ms [31, 41 ms], independent of the value of ω_TO_ (linear regression: *R*^2^=0.05, *F*=0.85, *P*=0.37).

To further interpret these results, we used Eqns 4 and 5a to simulate the rotational dynamics of spotted lanternfly nymphs during the mid-air phase of jumping for *n*=8 representative trials (1 trial per specimen) for which rotational kinematics were tracked and for which we were able to determine the angular velocity vector at take-off. For the interval of time, *t*_ext_=33 ms (16–49 ms) (mean, full range), over which the moment of inertia changed due to leg extension, **I**_rot_(*t*) was estimated by linearly interpolating the inertia tensor along each principal axis between those corresponding to each posture's 3D models using:
(8)

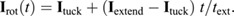


The resulting time-dependent inertia tensor was used to estimate effects due to posture changes resulting from changes in the principal axis directions and values of *I*_1_, *I*_2_, *I*_3_. The simulated values of ω_cc_ versus time were then averaged over 25 ms for comparison with the video analysis (Fig. 5). Results of the Wilcoxon two-sample signed-rank test for paired values of tracked and simulated ω_cc_(*t*) indicated that the measured and simulated data did not differ significantly (*P*<0.014) (Table S3). The measured and simulated data exhibited a similar overall functional behavior (i.e. a decrease in ω_cc_ with increasing time modulated by a lower amplitude oscillatory component). The oscillation period measured from simulations (Fig. 5), *T*_osc_=32.0 ms [23.3, 40.8 ms], agreed with tracked values (29.9 ms [21.7, 37.1 ms]) for the simulated datasets (Wilcoxon ranked sum, *W*=7.0, *P*=0.63, *n*=5 trials, 1 trial per specimen), although the simulations did not reproduce the amplitude and phase of the oscillations accurately in most trials analyzed. They also were unable to account for the abrupt increase in angular speed before impact observed in 6 of 21 trials (Fig. S2).

### Landing behavior

**Fig. 6.**
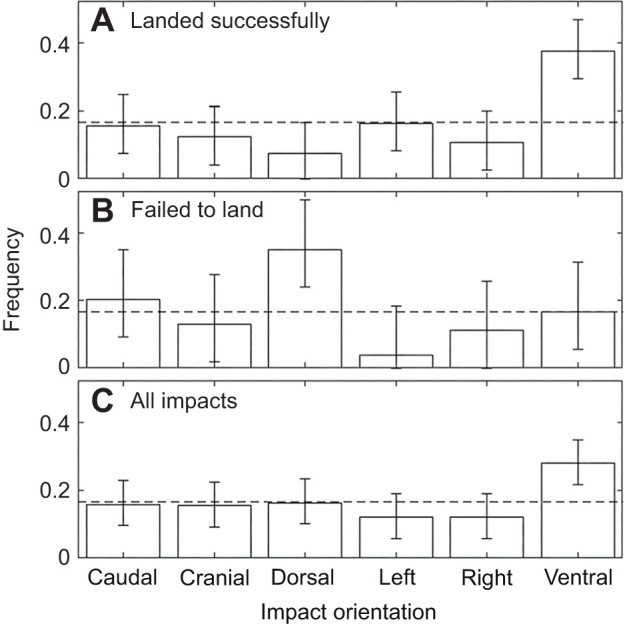
**Spotted lanternfly nymph body orientations at impact.** Data are shown for (A) all successful landings (*n*=122 landings, *N*=85 specimens, 1–4 landings per specimen), (B) all failed landing attempts (*n*=85 attempts, *N*=59 specimens, 1–4 attempts per specimen) and (C) all impacts. The error bars are the 95% confidence interval for each measured proportion. The dashed line at 1/6 corresponds to equal probability of landing in each orientation.

In spite of their rotational behavior in the air, spotted lanternfly nymphs were filmed landing securely on vegetation (outdoors) or on the target surface (in the laboratory) (Movie 1). A total of 207 videos filmed in the laboratory showed the body orientation relative to the surface on impact. Landing outcome data for the third and fourth instar nymphs were pooled because the distributions of impact orientation were not significantly different (chi-squared two-sample test: χ^2^=24, *P*=0.24 successful landings; χ^2^=18; *P*=0.26 failed landings). Significantly more specimens landed successfully than failed [probability of successful landing 58.9% [51.9, 65.7%]; binomial confidence interval, Clopper–Pearson method, *n*=207]. While specimens were observed landing successfully after impacting the surface at all possible orientations, successful landings were observed significantly more often than expected for impacts on the ventral surface, and failed landing attempts were significantly more frequent than expected for dorsal impacts (Fig. 6A,B). All other landing outcomes had no significant dependence on orientation. For all outcomes after impact, the ventral orientation was the most frequent and occurred significantly more frequently than expected (Fig. 6C).

While sometimes the spotted lanternfly nymphs were able to land securely upon first impact, there were multiple instances of them initially grasping the surface by only one or two tarsal claws and struggling to achieve a secure hold (Movie 1). The compliant fabric target appeared to absorb kinetic energy and allow the insects to grasp hold using their tarsal claws. A total of 25% (31/122) of videos showed specimens bouncing off the surface entirely at first impact but landing on a second try, either by sliding down the initial surface while trying to grasp it repeatedly or by bouncing onto and grasping another surface before reaching the ground. Some specimens performed a bellyflop landing, i.e. they first impacted on a side or the cranial or caudal end, then pivoted onto their ventral surface.

## DISCUSSION

The invasive spotted lanternfly is difficult to eradicate in part because of its agile jumping abilities. Our study revealed that spotted lanternfly nymphs regularly perform acrobatic jumps with complicated, but controlled, aerial rotations in the field and laboratory environments, with a high rate of landing successfully. In spite of their fast rotational motion, their jump trajectories agreed with zero drag force ballistic motion with performance measures similar to those reported for other planthoppers, providing no evidence of active control of their trajectory direction. Using photogrammetry, 3D modeling and simulation, we were able to reconstruct the complex, 3D rotational movements during a jump. This analysis also demonstrated how limb extension and subtle changes in limb position dramatically alter the moment of inertia and the principal axes of rotation, permitting fine adjustments to their body orientation.

While in the air, spotted lanternfly nymphs were found to spin rapidly (5–45 Hz) about rotational axes that varied in time relative to the body anatomical axes, consistent with the precessional motions predicted by 3D modeling. The main posture adjustments observed during jumping involved holding the legs tucked to varying degrees after take-off and then extending them rapidly 40–48 ms after take-off, comparable to measured insect neuromuscular response times (Sponberg and Full, 2008). Calculations using 3D modeling showed that this leg extension corresponds to ‘throws’ of body appendages (i.e. changes in both the moment of inertia and the body's principal axes of rotation) that led to complex changes in subsequent rotational motion (angular momentum twisting).

Using model parameters based on the angular velocity at take-off, two simplified 3D models of jumping postures, and drag torques based on fits to tracked orientation data, we were able to simulate the measured body orientation's angular speed, ω_cc_, versus time data so as to reproduce its overall dependence on time, the 11% decrease in ω_cc_ after the legs were fully extended, and the oscillations in ω_cc_ due to the time-varying direction of the axis of rotation resulting from precession immediately after take-off with a contribution from angular momentum twisting when the legs extend. The simulations showed that the rotational dynamics model needed to include the effect of aerodynamic drag torques to correctly describe the observed near-monotonic decrease in angular velocity during the mid-air phase. Modeling the configurations of the legs accurately using the 3D models was found to be important because they accounted for 61–82% of the moment of inertia values. The dominant contribution of the legs to rotational inertia means that changing leg extension causes large changes in the moment of inertia and the principal axes of rotation, and therefore in both rotational speed and axis direction (Table 3; Table S3). The model simulations therefore needed to include the change in leg extension at *t*_ext_ in order to accurately model the angular speed versus time. This also means that when the insect rotates its legs, this causes a larger counter-rotation of the body, enabling it to make attitude adjustments with fine corrections to the extension and orientation of its legs. Therefore, spotted lanternfly nymphs can adjust their axis of rotation, and hence body orientation, by flexing, extending or rotating their legs in mid-air to achieve attitude control.

In spite of their rapid rotational motion, spotted lanternfly nymphs were observed to be able to land securely in 58.9% of impacts. The results from rotational dynamic simulations indicate that their observed body posture changes can contribute to this ability. In addition, non-streamlined objects moving in the spotted lanternfly nymph's aerodynamic regime tend to orient with the maximum area normal to the airflow direction (Bennet-Clark, 1980; Vogel, 2020). Because our 3D models found the dorsal plane has the maximum area during both jumping postures, this suggests that aerodynamic drag torques promote orientations with either the ventrum or dorsum facing the airflow. The finding that specimens impacted on their ventral surfaces significantly more often than predicted by chance supports their use of attitude control to orient their ventrum facing the airflow, as previously reported for various insect species during falling (Ribak et al., 2013; Yanoviak et al., 2010; Zeng et al., 2017) and jumping (Burrows et al., 2015).

While landing attempts were disproportionately successful for impact on the ventral surface, spotted lanternfly nymphs were able to land securely for all orientations at impact. They did so by grasping the surface by one or more tarsi while rotating rapidly and bouncing, similar to the capabilities observed for falling spotted lanternfly nymphs landing on leaves (Kane et al., 2021) and for frogs landing on sticks (Bijma et al., 2016). This ability is of particular importance in their natural environment, where foliage landing spots present themselves at all orientations and locations, the ground is likely covered with clutter, and many landing targets recoil on impact. Thus, landing success can be enhanced by a number of behaviors, including achieving a preferred orientation when possible, but also by negotiating the aftermath of collisions so as to cling to the target (Jayaram et al., 2018; Kane et al., 2021; Reichel et al., 2019; Siddall et al., 2021).

The active control of body posture during the aerial phase of jumping also serves functions other than rotation and attitude control. For example, spotted lanternfly nymphs displaced by predators or disturbances benefit from landing on or near their current host plant in order to retain protective cover, avoid expending energy on finding and climbing a new host, and resume feeding. This is facilitated by extending their legs to increase the likelihood of encountering leaves while in the air. To estimate this effect, consider that the cross-section for encountering an obstacle can be estimated from the area of the minimum polygon enclosing the relevant body plane; these mean areas are 130 and 180 mm^2^ for the legs-tucked and -extended models, respectively, giving a 38% increase in area due to leg extension. Furthermore, because these insects often land using the same bellyflop method observed for frogs landing on sticks (Bijma et al., 2016), extending their legs to facilitate this landing method also could dissipate energy on impact to help stabilize landings. Furthermore, the mid-air posture during jumping differs from that observed for falling arthropods (Kane et al., 2021; Ribak et al., 2013; Yanoviak et al., 2015) in that spotted lanternflies, like planthoppers, leafhoppers, treehoppers, fleas and locusts (Burrows, 2009, 2011, 2013, 2014a,b; Burrows and Dorosenko, 2017; Burrows and Sutton, 2008; Burrows et al., 2007), cross their hindlegs in the leg-extended pose. Because the crossed hindlegs function as a single unit on impact, this configuration also might serve as bracing to provide protection against buckling on impact (Burrows and Sutton, 2008; Parle et al., 2015). Given that spotted lanternfly nymphs were able to keep their hindlegs crossed even after impact, it would be interesting to investigate their leg morphology for a latching mechanism, similar to the interlocking hairs or gear-like mechanism that some planthoppers use to synchronize leg motion (Burrows and Sutton, 2013).

These finding suggest multiple directions for future work. During take-off, a net torque can result from asymmetrical force production by the hindlegs (Burrows and Sutton, 2013) or from extending the body COM forward of the hindlegs’ push-off surface (Burrows, 2012). This indicates that rotations are likely for insects taking-off from flexible stems and leaves with varying orientations, as observed in our field videos. Consequently, it would be interesting to investigate how taking-off and landing behaviors differ for leaves, branches and solid ground with differing compliance and textures (Burrows and Sutton, 2008; Mo et al., 2020a; Ribak et al., 2012), as well as the effect of wind speed and orientation (Aguilar-Arguello et al., 2021; Wolfin et al., 2019), and to expand on prior modeling of take-off mechanisms relevant to tumbling control (Cofer et al., 2010).

Moreover, while field studies of spotted lanternflies have found that adults tend to land on vertical, high-contrast structures such as posts (Baker et al., 2021; Wolfin et al., 2019; Zeng et al., 2020), this possibility has not been investigated for nymphs. While spotted lanternfly nymphs did not show evidence of steering in this study, they were orienting toward a uniform target a relatively short distance away (0.21±0.06 m, mean±s.d.) compared with their mean jumping distances. Given that many other insects are known to steer toward preferred landing targets [e.g. leafhoppers (Brackenbury, 1996), flea beetles (Brackenbury and Wang, 1995) and stick insect nymphs (Zeng et al., 2015)], future work should consider the trajectories of jumping in naturalistic settings to see whether these insects exhibit targeted mid-air descent over their entire jumping distances.

While the results of this study demonstrate the utility of 3D modeling for use in simulating rotational dynamics in biomechanics, we only considered two characteristic postures observed during jumping by spotted lanternfly nymphs and transitions between these postures. The models presented here therefore were not intended to capture the effect of differences between individual body morphology or smaller changes in posture due to finer-scale leg adjustments during jumping that may also influence attitude control. In future work, it would be interesting to model the effect of leg motions in greater detail. One approach would be to obtain higher resolution video images of the insects to allow tracking of multiple body key points to determine body posture and 3D orientation in detail. Another would be to perform simulations using realistic 3D rendered models to inform the construction of articulated models with accurate mechanical properties but more easily simulated geometries (e.g. with cuboidal bodies and jointed rods for legs and appendages), similar to the jointed-rod model used successfully in earlier work (Burrows et al., 2015; Zeng et al., 2017).

The interplay between the complex jumping behavior of insects can yield new insights for animal behavior and for bioinspired jumping robotics. The approaches used here provide new insights into the physical mechanisms at work during aerial reorientation, and can easily be generalized to other organisms and motions.

## Supplementary Material

10.1242/jexbio.246340_sup1 Supplementary information
