# Supplementary material for: Putting a new spin on insect jumping performance using 3D modeling and computer simulations of spotted lanternfly nymphs
Source: J Exp Biol. 2023 Oct 6;226(19):jeb246340. doi: 10.1242/jeb.246340 (PMC10565111; doi:10.1242/jeb.246340)
Supplement: Supplementary information [file jexbio-226-246340-s1.pdf]

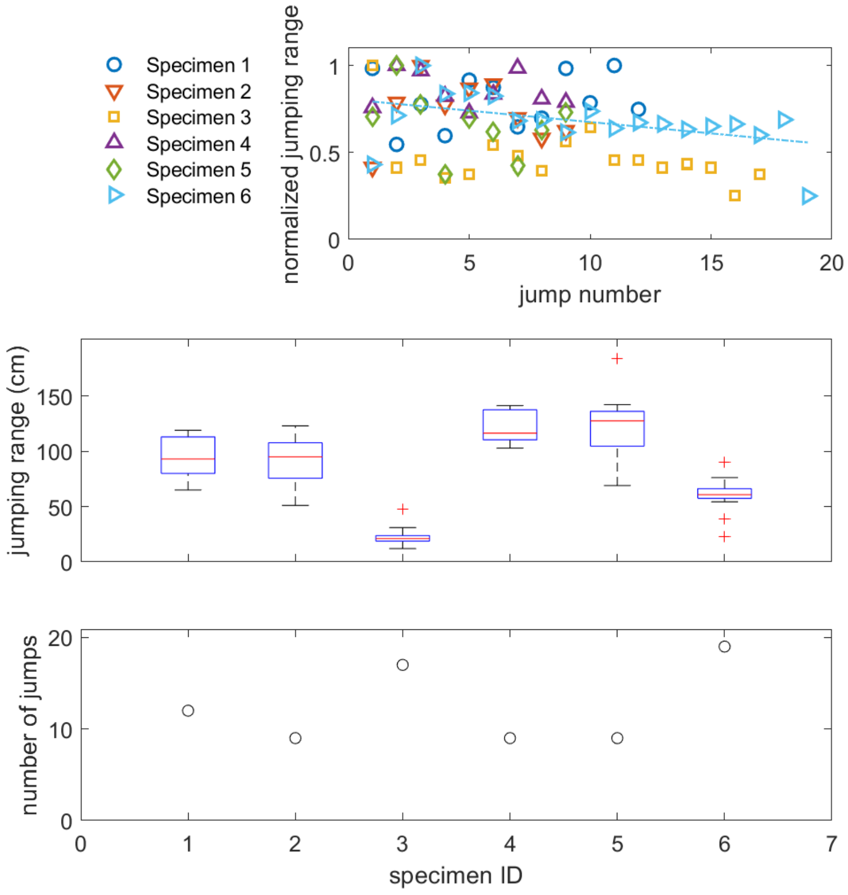

Fig. S1. Summary statistics from measurements of 4th instar spotted lanternfly nymph jumping fatigue measurements.

Journal of Experimental Biology • Supplementary information

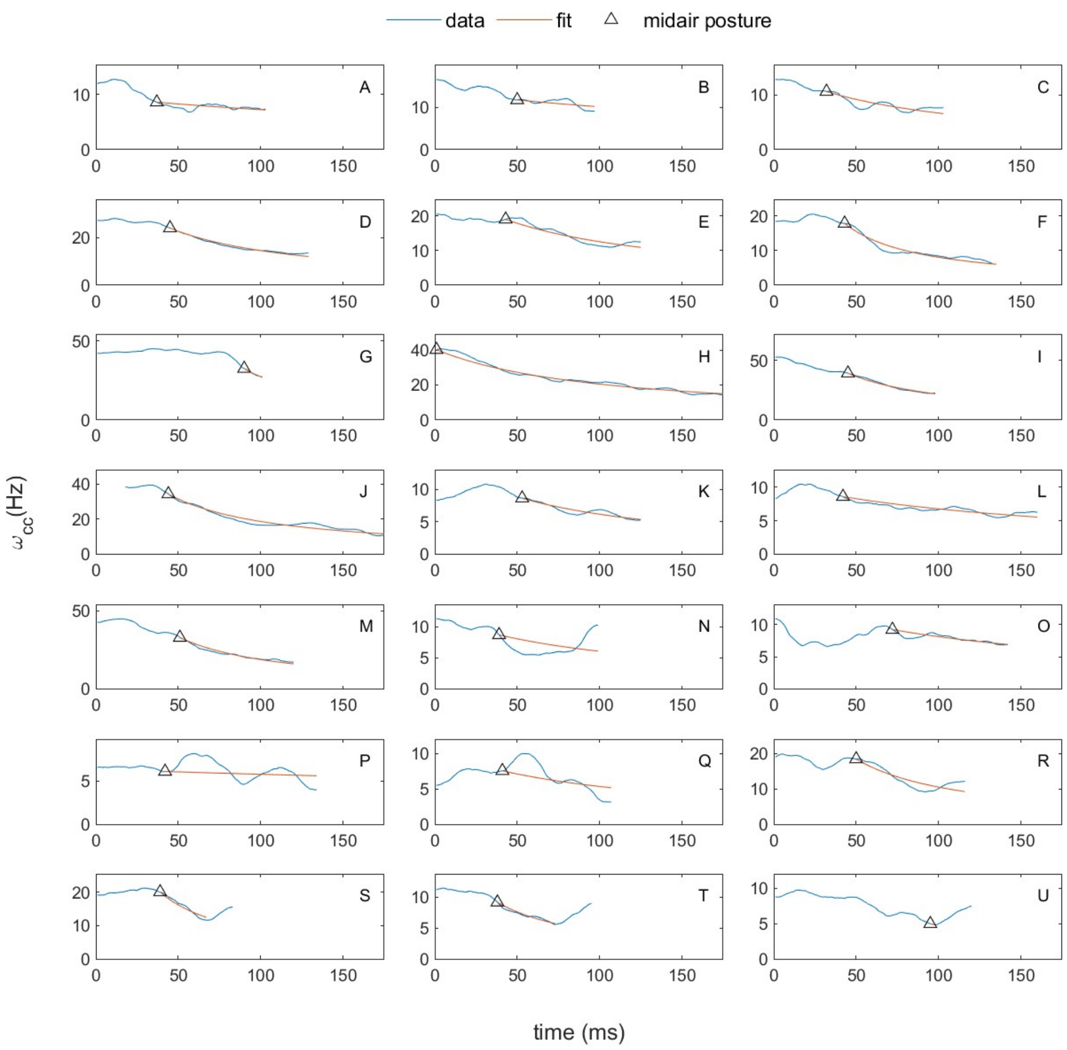

Fig. S2. Angular velocity of a spotted lanternfly nymph's body orientation (cranial-caudal body axis), ω_{cc}, vs time measured from video. (Blue line: angular velocity measured from video; red line: fit to Eq. 5b for rotational drag torque; time = 0 s at take-off; triangle markers = time at which legs were extended). Table S6 gives correspondences with the data shown in Fig. 5.

Journal of Experimental Biology • Supplementary information

Table S1. Results of linear regression performed on jumping distance normalized to the maximum value for each individual 4th instar spotted lanternfly nymph, R_{norm}, vs jump number in the sequence, N_{jump}. For the one specimen (6) with a significant dependence, the best line fit was R_{norm} = 0.81 [0.66, 0.95] – 0.013 [-0.026, -0.0005] * N_{jump} .

Specimen	R-squared	F-statistic	P-value	Degrees of freedom
1	0.04	0.46	0.51	10
2	0.004	0.030	0.87	7
3	0.20	3.78	0.07	15
4	0.033	0.24	0.64	7
5	0.13	1.0	0.34	7
6	0.22	4.8	0.042	17

Table S2. Fitting statistics for zero-drag ballistic model fits. The root-mean-squared (rms) residuals for the in-plane ballistic trajectory fits are: $e_{ballistic}$ (entire trajectory), $e_{take\text{-}off}$ (within 25 ms after take-off), e_{midair} (within 25 ms after midair posture changes), and 3) e_{impact} (within 5 ms of impact). R-squared is for the ballistic in-plane trajectory. The rms residual from the entire trajectory planar fit is e_{planar}. Values for $e_{take\text{-}off}$, e_{midair}, and e_{impact} were computed for all videos for which insects landed successfully without initially bouncing with a minimum time-of-flight of 55 ms, and hence have a different number of N (specimens) and n (trials) than the whole trajectory fits. For comparison, the 3D reconstruction calibration error is 0.54 mm. Data are given as grand means [95% CI].

Life stage	N	n	$e_{ballistic}$ (mm)	R-squared	e_{planar} (mm)	N	n	e_{midair} - $e_{take\text{-}off}$ (mm)	e_{impact}- e_{midair} (mm)
3rd instar	15	23	0.39 [0.31, 0.47]	0.9988 [0.9974 , 1.000]	0.45 [0.37, 0.53]	7	8	-0.11 [-0.21, 0.00]	0.17 [-0.03, 0.38]
4th instar	34	60	0.43 [0.35, 0.51]	0.9972 [0.9945, 0.9999]	0.48 [0.42, 0.54]	16	19	-0.02 [-0.08, 0.03]	-0.01 [-0.08, 0.06]

Journal of Experimental Biology • Supplementary information

Table S3. Results of Wilcoxon exact signed rank sum test on the tracked and simulated angular velocity data shown in Fig. 6

Fig. 5	Fig. S4	W test statistic	p-value
A	B	1441	0.001
B	F	7037	< 0.001
C	J	12346	< 0.001
D	M	4852	< 0.001
E	N	1732	0.014
F	R	6173	< 0.001
G	T	3188	< 0.001
H	C	717	< 0.001

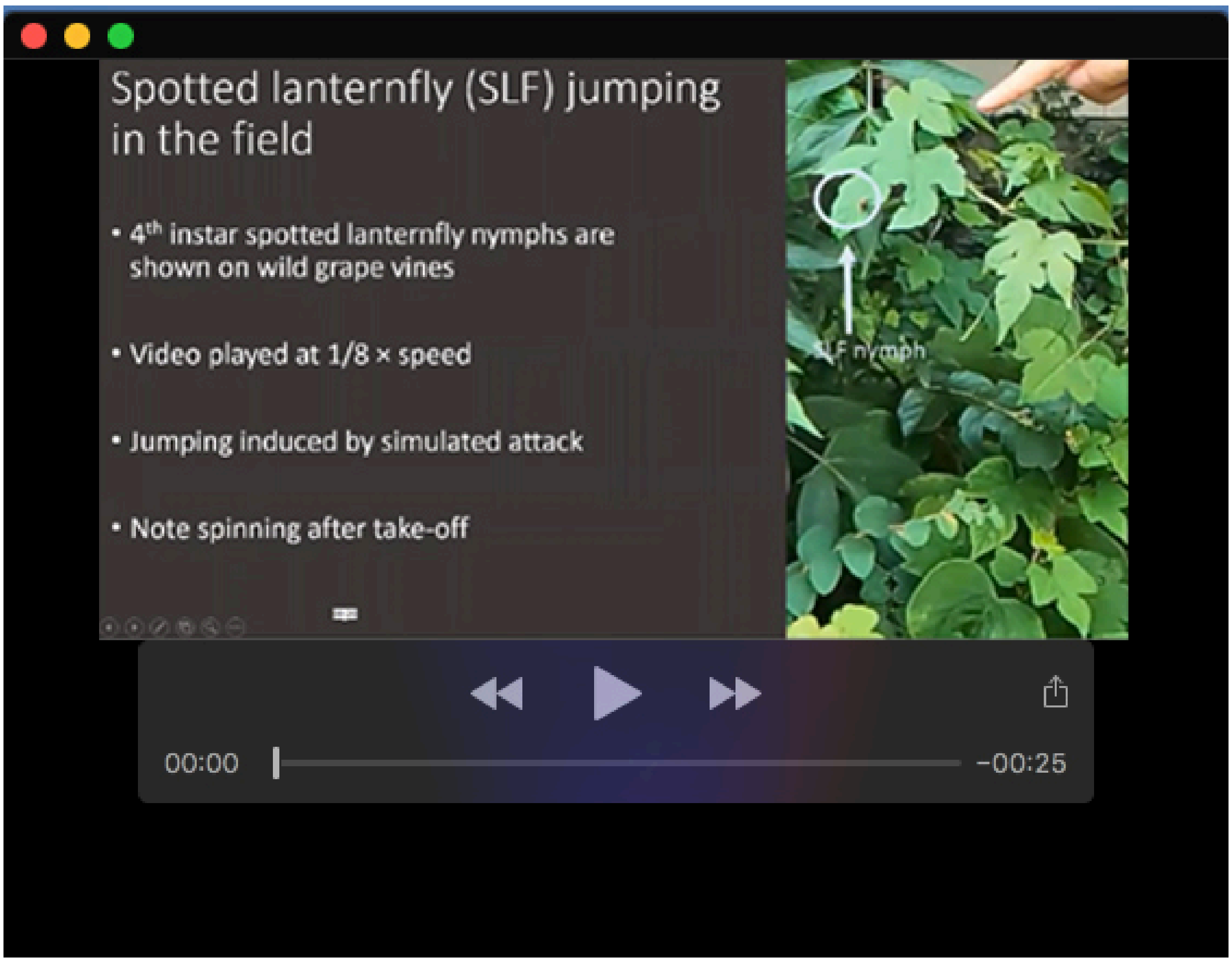

Movie 1. Example videos of spotted lanternfly nymphs jumping and landing in the field and laboratory; comparison of animations and experimental videos of the rotational dynamics during the midair phase of jumping trajectories.

Journal of Experimental Biology • Supplementary information
